# Problems and Barriers Related to the Use of Digital Health Applications: Protocol for a Scoping Review

**DOI:** 10.2196/32702

**Published:** 2022-04-21

**Authors:** Godwin Denk Giebel, Christian Speckemeier, Carina Abels, Kirstin Börchers, Jürgen Wasem, Nikola Blase, Silke Neusser

**Affiliations:** 1 Institute for Healthcare Management and Research University of Duisburg-Essen Essen Germany; 2 QM BÖRCHERS CONSULTING+ Herne Germany

**Keywords:** digital health application, DHA, mHealth, problems, barriers, scoping review, mobile health, health insurance, electronic database, health database, mHealth app

## Abstract

**Background:**

The use of mobile health (mHealth) apps is increasing rapidly worldwide. More and more institutions and organizations develop regulations and guidelines to enable an evidence-based and safe use. In Germany, mHealth apps fulfilling predefined criteria (Digitale Gesundheitsanwendungen [DiGA]) can be prescribed and are reimbursable by the German statutory health insurance scheme. Due to the increasing distribution of DiGA, problems and barriers should receive special attention.

**Objective:**

This study aims to identify the relevant problems and barriers related to the use of mHealth apps fulfilling the criteria of DiGA.

**Methods:**

This scoping review will follow published methodological frameworks and the PRISMA-Scr (Preferred Reporting Items for Systematic Reviews and Meta-analyses Extension for Scoping Reviews) criteria. Electronic databases (MEDLINE, EMBASE, PsycINFO, and JMIR), reference lists of relevant articles, and grey literature sources will be searched. Two reviewers will assess the eligibility of the articles by a two-stage (title and abstract as well as full text) screening process. Only problems and barriers related to mHealth apps fulfilling the criteria of DiGA are included for this research. The identified studies will be categorized and analyzed with MAXQDA.

**Results:**

This scoping review gives an overview of the available evidence and identifies research gaps regarding problems and barriers related to DiGA. The results are planned to be submitted to an indexed, peer-reviewed journal in the first quarter of 2022.

**Conclusions:**

This is the first review to identify the problems and barriers related to the use of mHealth apps fulfilling the German definition of DiGA. Nevertheless, the findings can be applied to other contexts and health care systems as well.

**International Registered Report Identifier (IRRID):**

DERR1-10.2196/32702

## Introduction

The use of mobile health (mHealth) apps becomes more and more ubiquitous. Health care systems worldwide establish different regulatory frameworks to integrate them into care. mHealth apps are used for many different purposes. For example, app-based vital signs monitoring can serve for (primary) prevention [1], medical adherence apps can lead to more regular medication intake [2], and other apps support coping with chronic diseases such as diabetes [3-5] or mental health problems [6,7].

According to the World Health Organization, digital health solutions can contribute to higher standards of health and better access to health care services. The use of digital health allows people worldwide to promote and protect their health and well-being [8]. While opportunities and possibilities are considered certain, the problems and barriers related to the use of mHealth apps often remain unaddressed.

Realizing the potential benefits of digitalization, the national Parliament of the Federal Republic of Germany (Deutscher Bundestag) introduced “the Act to Improve Healthcare Provision through Digitalization and Innovation (Digital Healthcare Act – DVG)” in 2019 [9]. One of the main innovations is the possibility to prescribe predefined mHealth apps, called Digitale Gesundheitsanwendungen (DiGA). These prescribed apps are reimbursable for 73 million people insured under the German statutory health insurance scheme.

Focusing on the following requirements, this text applies the term “Digital Health Application” (DHA) to describe mHealth apps that meet the criteria for DiGA.

In Germany, mHealth apps must fulfill a predefined list of criteria to be regarded as a DiGA [10]. These criteria are as follows: (1) medical device of a risk class lower or equal to IIa; (2) the main function of the app is based on digital technology; (3) the DHA is not only a medium to collect data from a device or to control a device. The app must have a proper main digital function achieving a medical purpose; (4) recognition, monitoring, treatment or alleviation of diseases, or the recognition, treatment or alleviation or compensation of injuries are supported by the DiGA; (5) the purpose of the app is not for primary prevention; and (6) the app is used either by the patient or by the health care provider together with the patient. Therefore, apps only used by health care providers are not assessed as DiGA. DiGA can be considered as “digital assistants” in the hands of patients.

Currently, there are 28 certified DHAs listed in the German DiGA registry, covering a range of indications including mental illnesses such as depression, anxiety disorder, or addiction, as well as physical illnesses such as arthrosis, multiple sclerosis, and diabetes mellitus.

The German DiGA concept is unique, though other countries are considering launching a similar concept. In particular, France has announced to adopt the German system. Therefore, the German system might serve as a blueprint for other countries regarding the integration of DHA in their health care systems.

Reviews on the evidence of problems and barriers related to the use of DHA are sparse. While O’Connor et al [11] identified and synthesized the qualitative literature on barriers and facilitators to engagement and recruitment to digital health interventions [11], Kao and Liebovitz [12] present the current state, barriers, and future directions of consumer mHealth apps. Besides the broad reviews mentioned before, there is a qualitative study by Stiles-Shields et al [13] investigating the barriers to the use of apps for depression. Finally, Meyerowitz-Katz [14] investigated the rates of attrition and dropout in app-based interventions for chronic disease. In their review and meta-analysis, they rather focused on dropout rates in apps for chronic disease than reasons for dropping out.

Ahmad et al [15] planned a scoping review to capture current problems and opportunities in the adoption of mobile apps among older adults. Contrary to this scoping review, our review does not exclude younger users.

The only review focusing specifically on DiGA is part of the German Advisory Council on the Assessment of Developments in the Health Care System report [16]. It focusses on evidence but not on barriers or problems in the context of DiGA.

Considering this research gap, the planned scoping review aims to analyze the following research question: “which problems and barriers related to the use of mHealth apps comparable to the German DiGA concept are addressed in studies?”

The scoping review is part of a wider research project (continuous quality assurance of DHA [QuaSiApps]) funded by the Federal Joint Committee (G-BA) [17].

## Methods

### Guidance Frameworks

The scoping review will be conducted according to the framework with the 5 (mandatory) stages described by Arksey and O’Malley [18] in 2005 and further developed by Levac et al [19] in 2010. These stages are as follows: (1) identifying the research question, (2) identifying relevant studies, (3) study selection, (4) charting the data and collating, and (5) summarizing and reporting the results. Following these stages will guarantee a systematic and coherent proceeding. The subsequent preparation of the manuscript will follow the PRISMA-Scr (Preferred Reporting Items for Systematic Reviews and Meta-analyses Extension for Scoping Reviews) by Tricco et al [20]. The protocol was not registered.

### Stage I: Identifying the Research Question

The research question, “Which problems and barriers related to the use of mHealth apps comparable to the German DiGA concept are addressed in studies?” was posed. This scoping review will provide a structured overview for further research and inform stakeholders. Furthermore, it serves as one module of our research project, QuaSiApps.

### Stage II: Identifying Relevant Studies

The search strategy is predefined according to the Joanna Briggs Institute Manual for Evidence Synthesis [21]. After an initial explorative research and subsequent team discussion, terms and keywords were determined and used to conduct the main search across the included databases (EMBASE, MEDLINE, PsycINFO, and JMIR). Afterward, the reference lists of included studies will be screened, and referenced articles assessed according to the predefined inclusion and exclusion criteria. If they are appropriate, they will be included in the scoping review.

The search was performed following Methodology, Issues, Participants (MIP) [22] scheme including methodology (all methodologies), issues (problems and barriers related to the use of DHA), and participants (focus on patients and health care providers).

Electronic databases EMBASE, MEDLINE, and PsycINFO were searched on June 8, 2021. The used search terms were combined in the following manner: (*”difficulty“* OR *”obstacle“* OR *”problem“* OR *”issue“* OR *”challenge“* OR *”barrier“*) AND (*”web application“* OR *”mobile application“* OR *”mHealth“* OR *”virtual care“* OR *”healthcare app“* OR *”health care app“* OR *”mobile health“* OR *”health app“*) OR (*”smartphone“* OR *”mobile phone“* OR *”android“* OR *”iphone“* OR *”browser“* AND *”health“*) AND (*”healthcare“* OR *”health care“*).

The respective search terms were restricted to the occurrence in abstract, title, or keyword but expanded by indexing terms (Medical Subject Headings [MeSH] and Emtree). The complete search strategy can be found in Multimedia Appendices 1-3. Due to the thematic focus of JMIR, we added a structured search using the search function as well as relevant themes.

Searching the two databases EMBASE and MEDLINE and carrying out structured research in JMIR provide points of view of many different disciplines, which was deemed necessary in order to depict the multidisciplinary field of mHealth apps. In addition, PsycINFO was searched because a large proportion of certified DiGA in Germany stems from the field of mental illnesses.

Language was restricted to English, German, and French. The research was limited to articles published between January 1, 2015, and June 8, 2021. Further explanation for time restriction is given in the discussion of this protocol.

Apart from the online database research, gray literature sources such as the Federal Institute for Drugs and Medical Devices reports, guidelines, working papers, and industry reports will be searched via institutional websites and Google search engine (Multimedia Appendix 4).

### Stage III: Study Selection and Eligibility Criteria

In the first step, the identified citations were uploaded in the literature management program Endnote X9 (Clarivate Analytics), and duplicates were removed. In the second step, 2 reviewers (GG and CS) will decide whether an article is eligible for full-text screening by independently assessing the title and abstract. In a third step, the full-text screening of the included articles and assessment against the exclusion criteria will be conducted by the same 2 reviewers. In this third step, the reasons for excluding studies will also be captured.

Disagreement between the 2 reviewers during the screening process will be resolved through discussion. If necessary, a third person (SN) will resolve emerging conflicts. In case of missing data, the reviewers will contact the authors of the included papers.

To handle the vast magnitude of mHealth apps and to balance between breadth and feasibility, we defined exclusion criteria, which we adjusted in the initial search process. Our final inclusion and exclusion criteria are listed in Textbox 1.

**Inclusion and exclusion criteria.**

**Inclusion criteria**

- Articles mentioning problems and barriers related to the use of mobile health apps
- A problem term mentioned in the abstract or title was related to the use of mobile health apps
- Publication with focus on mobile health apps
- Examined mobile health apps fulfill the requirements set for Digital Health Application (Digitale Gesundheitsanwendungen)
- Article published in 2015 or afterward
- Language: English, German, or French

**Exclusion criteria**

- Not valid to answer the research question
- The problem term mentioned in the abstract or title was not related to mobile health apps
- Publication does not focus on mobile health apps.
- Examined mobile health apps fulfill one or more of the following criteria:
- Not used by the patient
- No relation to illness, injury, or handicap
- Primary prevention
- The medical purpose is not achieved through the main digital functions
- Research protocol or conference abstract
- Article published before 2015
- Language other than English, German, or French

### Stage IV: Charting the Data

The remaining publications will be included in the scoping review. Relevant information and data such as authors, year, country, study type underlying diseases, and especially problems and barriers related to DHA will be extracted (Table 1).

**Table 1:** Data extraction.

Author	Year	Study type	Country	Underlying disease	Problems
					

### Stage V: Collating, Summarizing, and Reporting Results

Following the recommendations by Levac et al [19], the fifth stage is divided in the following three distinct steps: (1) analyzing research findings (including descriptive numerical summary analysis and qualitative thematic analysis); (2) evaluating the research findings and extracting an outcome that is in accordance with the research question (the results are reported in a narrative way); and (3) interpreting and discussing the findings with regard to further research questions, practice, and policy.

Besides the narrative reporting, tables and figures will ensure a structured overview about the key findings. The PRISMA-Scr [20] serves to guarantee a systematic reporting of the results.

## Results

A coherent search strategy to identify articles focusing on problems and barriers related to the use of DHA and DiGA was developed. The results of our investigation will be presented and published in a systematic scoping review. Therefore, the process of publication selection will be presented using flowcharts, and the extracted data of our research will be systematized in tables and described in a narrative summary.

Data synthesis will not follow the existing themes. Problems and barriers related to the use of DiGA will be grouped in categories defined by the authors. If a problem does not fit to a defined group of problems, a new group will be created. Subsequently, the results and parent categories of problems aim to answer the research question, “Which problems and barriers related to the use of mHealth apps comparable to the German DiGA concept are addressed in studies?”

## Discussion

There is a multitude of mHealth apps in nearly every domain of medicine. The opportunities and possibilities of mHealth apps are often discussed, whereas the research on related problems and barriers remain scarce. This scoping review will fulfill two reasons for conducting scoping reviews according to Arksey and O’Malley [18]. First, it summarizes and disseminates research findings to policy makers, practitioners, and consumers. Second, it allows researchers and other stakeholder to identify research gaps in the existing literature.

Our scoping review has some limitations, which cannot be prevented due to resource limitation. The research is restricted to articles published after the year 2015. Evidence gained before the time restriction is only captured if it is incorporated in newer publications. Two facts made the year 2015 a reasonable starting point for the scoping review. On the one hand, previous reviews show that older investigations in the context of mHealth cover mainly text messaging interventions [23]. This was found, for example, for the effectiveness of mHealth interventions focused on health care workers to improve pregnancy outcomes in low- and middle-income countries [24] or the use of mHealth in antenatal and postpartum care and vaccination administration [25]. Text messaging apps do not provide the criteria to be considered as a DiGA. On the other hand, in 2015, Stoyanov et al [26] published the Mobile App Rating Scale (MARS). The MARS is the first tool to assess app quality, which is in direct conjunction with DHA problems.

Further limitations arise from the rather new and rapid advancing technology of mHealth, which also hamper a systematic search. Moreover, the terminology in the field of mHealth apps is not consistent. Even in the most known publications (eg, Mobile “App” Rating Scale [26] and Mobile “Application” Rating Scale [27]), there is no consistency in terminology. While some institutions such as the German Federal Institute for Drugs and Medical Devices use the term “(Digital Health-) Application,” a consensus paper recommends the use of “app” instead of “application” [28]. When constructing our search strategy, different terms were pilot tested, and results were compared. In addition, the inclusion of related terms ensures a broad coverage of the topic.

Nevertheless, there still is uncertainty whether all relevant search terms might be covered. This uncertainty also applies to the selection of databases. Medline, Embase, and PsychINFO, the most important databases for classical medical and psychological therapies and devices, are covered. However, these might not cover all relevant journals in the rapidly evolving field of mHealth, and we addressed these limitations by conducting a structured search through JMIR.

A further limitation is made due to the context of the scoping review. The scoping review is one module of the larger research project, QuaSiApps, which aims to develop a generic quality assurance system. Therefore, we could not restrict the research to specific diseases. In order to guarantee research specific to our study and exclude lifestyle, wellness, and fitness apps, we restricted our search to health care.

Exclusion criteria ensured that publications were only included if problems related to mHealth apps were mentioned in the title or the abstract. Other publications describing mHealth apps could include problems or barriers as a secondary aspect. Nevertheless, publications with problems as a central subject are covered.

### Conclusion

This scoping review will be the first that provides an overview about potential problems and barriers related to the use of DHA according to the German definition of DiGA. The research findings of this and a further scoping review about the quality of DHA will serve as a first module for the development of a continuous quality assurance concept [29]. In the next step, we will use the research findings to develop a discussion guide to conduct focus groups with users and potential users of DHA.

## Appendix

Multimedia Appendix 1 Search strategy; Medline via Embase.

Multimedia Appendix 2 Search strategy; Medline via Ovid.

Multimedia Appendix 3 Search strategy; PsycINFO via Ovid.

Multimedia Appendix 4 Sources of gray literature.

## Abbreviations

DHA: Digital Health Application
DiGA: Digitale Gesundheitsanwendungen
MeSH: Medical Subject Headings
mHealth: mobile health
MIP: Methodology, Issues, Participants
PRISMA-Scr: Preferred Reporting Items for Systematic Reviews and Meta-analyses Extension for Scoping Reviews
QuaSiApps: continuous quality assurance of DHA
