# Supplementary material for: Problems and Barriers Related to the Use of Digital Health Applications: Protocol for a Scoping Review
Source: JMIR Res Protoc. 2022 Apr 21;11(4):e32702. doi: 10.2196/32702 (PMC9073601; doi:10.2196/32702)
Supplement: Multimedia Appendix 1 [file resprot_v11i4e32702_app1.docx]

**Multimedia Appendix 1.** Search strategy; Medline via Embase.

| Database | EMBASE | |
| --- | --- | --- |
| Platform | **Embase.com** | |
| Date of search | **08.06.2021** | |
| Filter | **No filters** | |
| # | Search Term | Hits |
| 1a | difficulty:ab,ti,kw | 174,788 |
| 1b | obstacle:ab,ti,kw | 29,095 |
| 1c | problem:ab,ti,kw | 679,836 |
| 1d | issue:ab,ti,kw | 330,179 |
| 1e | challenge:ab,ti,kw | 480,528 |
| 1f | barrier:ab,ti,kw | 240,189 |
| 1g | **(1a + 1b + 1c + 1d + 1e + 1f)** | **1,846,644** |
|  |  |  |
| 2a.1 | 'web application':ab,ti,kw | 2,955 |
| 2a.2 | 'web application'/exp/mj | 6 |
| 2a | **'web application':ab,ti,kw OR 'web application‘/exp/mj** | **2,957** |
| 2b.1 | 'mobile application':ab,ti,kw | 3,193 |
| 2b.2 | 'mobile application'/exp/mj | 8,163 |
| 2b | **'mobile application':ab,ti,kw OR 'mobile application'/exp/mj** | **9,825** |
| 2c.1 | mHealth:ab,ti,kw | 5,968 |
| 2c.2 | mHealth/exp/mj | 23 |
| 2c | **mHealth:ab,ti,kw OR mHealth/exp/mj** | **5,969** |
| 2d.1 | 'virtual care':ab,ti,kw | 453 |
| 2d.2 | 'virtual care'/exp/mj | 11 |
| 2d | **'virtual care':ab,ti,kw OR 'virtual care'/exp/mj** | **455** |
| 2e.1 | 'healthcare app':ab,ti,kw | 14 |
| 2e.2 | No Emtree |  |
| 2e | **'healthcare app':ab,ti,kw** | **14** |
| 2f.1 | 'health care app':ab,ti,kw | 13 |
| 2f.2 | No Emtree |  |
| 2f | **'health care app':ab,ti,kw** | **13** |
| 2g.1 | 'mobile health':ab,ti,kw | 5,666 |
| 2g.2 | 'mobile health'/exp/mj | 99 |
| 2g | **'mobile health‘:ab,ti,kw OR 'mobile health'/exp/mj** | **5,696** |
| 2h.1 | 'health app':ab,ti,kw | 391 |
| 2h.2 | No Emtree |  |
| 2h | **'health app':ab,ti,kw** | **391** |
| 2i | **(2a + 2b + 2c + 2d + 2e + 2f + 2g +2h)** | **20,032** |
|  |  |  |
| 3a.1 | smartphone:ab,ti,kw | 16,159 |
| 3a.2 | smartphone/exp/mj | 6,352 |
| 3a | **smartphone:ab,ti,kw OR smartphone/exp/mj** | **17,070** |
| 3b.1 | 'mobile phone':ab,ti,kw | 9,288 |
| 3b.2 | 'mobile phone'/exp/mj | 13,698 |
| 3b | **'mobile phone':ab,ti,kw OR 'mobile phone'/exp/mj** | **19,405** |
| 3c.1 | android:ab,ti,kw | 4,672 |
| 3c.2 | android/exp/mj | 1,385 |
| 3c | **android:ab,ti,kw OR android/exp/mj** | **1,387** |
| 3d.1 | iphone:ab,ti,kw | 1,771 |
| 3d.2 | iphone/exp/mj | 11 |
| 3d | **iphone:ab,ti,kw OR iphone/exp/mj** | **1,773** |
| 3e.1 | browser:ab,ti,kw | 4,737 |
| 3e.2 | No Emtree |  |
| 3e | **browser:ab,ti,kw** | **4,737** |
| 3f | **(3a + 3b + 3c + 3d + 3e)** | **35,703** |
|  |  |  |
| 4a | **health:ab,ti,kw** | **2,698,360** |
|  |  |  |
| 5a | **3f AND 4a** | **11,014** |
|  |  |  |
| 6a | healthcare:ab,ti,kw | 399,519 |
| 6b | 'health care':ab,ti,kw | 484,075 |
| 6c | **(6a + 6b)** | **842,951** |
|  |  |  |
| 7a | **1g AND (2i OR 5a) AND 6c** | **809** |

(difficulty:ab,ti,kw OR obstacle:ab,ti,kw OR problem:ab,ti,kw OR issue:ab,ti,kw OR challenge:ab,ti,kw OR barrier:ab,ti,kw)

AND

(('web application':ab,ti,kw OR 'web application‘/exp/mj OR 'mobile application':ab,ti,kw OR 'mobile application'/exp/mj OR mHealth:ab,ti,kw OR mHealth/exp/mj OR 'virtual care':ab,ti,kw OR 'virtual care'/exp/mj OR 'healthcare app':ab,ti,kw OR 'health care app':ab,ti,kw OR 'mobile health‘:ab,ti,kw OR 'mobile health'/exp/mj OR 'health app':ab,ti,kw OR 'health app'/exp/mj)

OR

((smartphone:ab,ti,kw OR smartphone/exp/mj OR 'mobile phone':ab,ti,kw OR 'mobile phone'/exp/mj OR android:ab,ti,kw OR android/exp/mj OR iphone:ab,ti,kw OR iphone/exp/mj OR browser:ab,ti,kw) AND health:ab,ti,kw))

AND

(healthcare:ab,ti,kw OR 'health care':ab,ti,kw)
