# Supplementary material for: Problems and Barriers Related to the Use of Digital Health Applications: Protocol for a Scoping Review
Source: JMIR Res Protoc. 2022 Apr 21;11(4):e32702. doi: 10.2196/32702 (PMC9073601; doi:10.2196/32702)
Supplement: Multimedia Appendix 3 [file resprot_v11i4e32702_app3.docx]

**Multimedia Appendix 3.** Search strategy; PsycINFO via Ovid.

| Database | PsycInfo | |
| --- | --- | --- |
| Platform | **Ovid** | |
| Date of search | **08.06.2021** | |
| Filter | **No filters** | |
| # | Search Term | Hits |
| 1a | difficulty.mp | 69,365 |
| 1b | obstacle.mp | 4,973 |
| 1c | problem.mp | 255,480 |
| 1d | issue.mp | 143,106 |
| 1e | challenge.mp | 81,563 |
| 1f | barrier.mp | 18,931 |
| 1g | **(1a + 1b + 1c + 1d + 1e + 1f)** | **538,692** |
|  |  |  |
| 2a.1 | "web application".mp | 200 |
| 2a.2 | No MeSH |  |
| 2a | **"web application".mp** | **200** |
| 2b.1 | "mobile application".mp | 652 |
| 2b.2 | exp "mobile application"/ | 1210 |
| 2b | **"mobile application".mp OR exp "mobile application"/** | **1565** |
| 2c.1 | mHealth.mp | 1183 |
| 2c.2 | exp mHealth/ | 1189 |
| 2c | **mHealth.mp OR exp mHealth/** | **1588** |
| 2d.1 | "virtual care".mp | 40 |
| 2d.2 | No MeSH |  |
| 2d | **"virtual care".mp** | **40** |
| 2e.1 | "healthcare app".mp | 0 |
| 2e.2 | No MeSH |  |
| 2e | **"healthcare app".mp** | **0** |
| 2f.1 | "health care app".mp | 0 |
| 2f.2 | No MeSH |  |
| 2f | **"health care app".mp** | **0** |
| 2g.1 | "mobile health".mp | 1565 |
| 2g.2 | exp "mobile health"/ | 1189 |
| 2g | **"mobile health".mp OR exp "mobile health"/** | **1565** |
| 2h.1 | "health app".mp | 80 |
| 2h.2 | No MeSH |  |
| 2h | **"health app".mp** | **80** |
| 2i | **(2a + 2b + 2c + 2d + 2e + 2f + 2g +2h)** | **3,398** |
|  |  |  |
| 3a.1 | smartphone.mp | 3,820 |
| 3a.2 | No MeSH |  |
| 3a | **smartphone.mp** | **3,820** |
| 3b.1 | "mobile phone".mp | 2,824 |
| 3b.2 | exp "mobile phone"/ | 5,921 |
| 3b | **"mobile phone".mp OR exp "mobile phone"/** | **7,193** |
| 3c.1 | android.mp | 519 |
| 3c.2 | No MeSH |  |
| 3c | **android.mp** | **519** |
| 3d.1 | iphone.mp | 302 |
| 3d.2 | No MeSH |  |
| 3d | **iphone.mp** | **302** |
| 3e.1 | browser.mp | 405 |
| 3e.2 | No MeSH |  |
| 3e | **browser.mp** | **405** |
| 3f | **(3a + 3b + 3c + 3d + 3e)** | **9,412** |
|  |  |  |
| 4a | **health.mp** | **739,105** |
|  |  |  |
| 5a | **3f AND 4a** | **2,632** |
|  |  |  |
| 6a | healthcare.mp | 49,974 |
| 6b | "health care".mp | 190,466 |
| 6c | **(6a + 6b)** | **220,232** |
|  |  |  |
| 7a | **1g AND (2i OR 5a) AND 6c** | **176** |

(difficulty.mp OR obstacle.mp OR problem.mp OR issue.mp OR challenge.mp OR barrier.mp)

AND

(("web application".mp OR "mobile application".mp OR exp "mobile application"/ OR mHealth.mp OR exp mHealth/ OR "virtual care".mp OR "healthcare app".mp OR "health care app".mp OR "mobile health".mp OR exp "mobile health"/ OR "health app".mp)

OR

((smartphone.mp OR "mobile phone".mp OR exp "mobile phone"/ OR android.mp OR iphone.mp OR browser.mp) AND health.mp))

AND

(healthcare.mp OR "health care".mp)
